# Computational Discovery of Dual-Target LDHA/BRD4 Inhibitors Targeting the Lactate–Kla–B7-H3 Immunosuppressive Axis Through AI-Driven Virtual Screening

**DOI:** 10.3390/ph19050736

**Published:** 2026-05-07

**Authors:** Ruiqi Zhao, Mengyao Han, Bei Zhang, Mengqing Ma, Xiaozhou Zhou, Jialing Sun

**Affiliations:** 1 The Fourth Clinical Medical College, Guangzhou University of Chinese Medicine, Shenzhen 518033, China; 20252120304@stu.gzucm.edu.cn (R.Z.);; 2 The Second Clinical Medical College, Guangzhou University of Chinese Medicine, Guangzhou 510405, China; 3 Faculty of Chinese Medicine, Macau University of Science and Technology, Taipa, Macao 999078, China

**Keywords:** virtual screening, artificial intelligence, deep learning, molecular dynamics simulations, quantum chemistry, hepatocellular carcinoma, LDHA, BRD4

## Abstract

**Background/Objectives:** Immune evasion remains a critical barrier to effective hepatocellular carcinoma (HCC) therapy. Lactate dehydrogenase A (LDHA) drives lactate accumulation and histone lysine lactylation (Kla), reshaping the immunosuppressive microenvironment, while bromodomain-containing protein 4 (BRD4) sustains B7-H3 transcription via super-enhancer occupancy. Despite their synergistic roles in the lactate–Kla–B7-H3 immunosuppressive axis, no dual-target inhibitor simultaneously engaging both proteins has been reported. This study aimed to discover dual LDHA/BRD4 inhibitors from natural product libraries using an integrated AI-driven computational pipeline. **Methods:** We established a multi-tier virtual screening cascade comprising Lipinski/QED drug-likeness filtration, DiffDock-based AI docking, QuickVina binding energy validation, PLIP interaction profiling, 200 ns all-atom molecular dynamics simulations, MM-GBSA binding free energy calculations, and density functional theory analysis. Natural product libraries from COCONUT and CMNPD databases (84,730 compounds post-filtration) were screened against both targets. **Results:** High-throughput DiffDock screening identified 11 dual-target hits, from which CNP0038114.1 and CMNPD16582 emerged as prioritized lead candidates. All four protein–ligand complexes maintained structural stability throughout MD simulations, with MM-GBSA binding free energies ranging from −27.24 to −32.45 kcal/mol, predominantly driven by van der Waals interactions. DFT calculations revealed distinct electronic profiles: CNP0038114.1 exhibited a narrow HOMO–LUMO gap (2.718 eV) favoring charge-transfer reactivity, whereas CMNPD16582 displayed a larger gap (4.822 eV), suggesting superior chemical stability. **Conclusions:** This computational study furnishes two novel natural product leads for targeting the lactate–Kla–B7-H3 immunosuppressive axis in HCC, establishing a generalizable AI-driven workflow for dual-target inhibitor discovery.

## 1. Introduction

Lactate dehydrogenase A (LDHA) and bromodomain-containing protein 4 (BRD4), serving as pivotal nodal proteins in metabolic reprogramming and epigenetic regulation respectively, have increasingly been implicated as central orchestrators of malignant progression and immune evasion in hepatocellular carcinoma (HCC) [1,2]. As the terminal rate-limiting enzyme of glycolysis, LDHA catalyzes the reductive conversion of pyruvate to lactate and is markedly overexpressed in HCC within the context of the Warburg effect, with its aberrantly elevated enzymatic activity directly precipitating massive lactate accumulation in the tumor microenvironment (TME) [3]. Compelling evidence has demonstrated that elevated intracellular lactate concentrations not only suppress the cytotoxic capacity of effector T cells and natural killer (NK) cells through microenvironmental acidification, but more crucially, lactate functions as an acyl donor to propel histone lysine lactylation (Kla)—a post-translational modification whose levels, particularly at the H3K18la site, are significantly upregulated in HCC tissues [4,5,6]. This emerging epigenetic mark has been shown to activate transcriptional programs governing an array of immunosuppressive genes, including those promoting M2-type macrophage polarization, thereby remodeling the tumor immunosuppressive microenvironment at the epigenetic level [7]. BRD4, the flagship member of the bromodomain and extra-terminal domain (BET) family, recognizes and engages acetylated lysine marks on histones through its tandem bromodomains (BD1 and BD2), subsequently recruiting the transcriptional elongation machinery to target gene promoters and super-enhancer (SE) loci to drive oncogene and immune checkpoint molecule transcription [8,9]. In HCC, BRD4 has been found to occupy the SE region of the B7-H3 (CD276) gene locus, directly sustaining its elevated transcriptional output. B7-H3, a member of the B7 family of immune checkpoint ligands, transmits co-inhibitory signals through interactions with receptors on T cells and NK cells, constituting a critical molecular barrier for HCC evasion of antitumor immunosurveillance [10,11].

Of particular note, LDHA and BRD4 exhibit functional convergence and mechanistic synergy within the HCC immunosuppressive network. On the one hand, LDHA-driven lactate accumulation elevates global Kla levels, indirectly potentiating the epigenetic activation state of immunosuppressive gene loci, including B7-H3; on the other hand, as an acetylation reader, the functional engagement of BRD4 at SEs may itself be modulated by the local chromatin lactylation landscape [12]. Consequently, LDHA and BRD4 cooperatively drive B7-H3 overexpression and the immune evasion phenotype from the metabolite supply and transcriptional activation axes, respectively, establishing a synergistic regulatory circuit. Theoretically, a dual-target inhibition strategy simultaneously engaging LDHA and BRD4 could achieve a superimposed therapeutic effect, dismantling the immunosuppressive barrier of HCC more effectively than single-target intervention alone. Nevertheless, although LDHA and BRD4 have each been validated as translationally promising anti-HCC drug targets, all currently available inhibitors for these two proteins are mono-target selective molecules with inherent pharmacokinetic limitations and resistance liabilities [13,14]. No small-molecule inhibitor capable of concomitantly engaging the LDHA catalytic pocket and the BRD4 bromodomain acetyl-lysine recognition site has been reported, and this dual-target molecular design challenge renders conventional single-target drug discovery strategies inapplicable, necessitating the exploration of novel methodological avenues for the de novo identification of dual-target active molecules.

Computer-aided virtual screening has now emerged as a powerful engine for new drug discovery. Conventional high-throughput screening, reliant on physical compound libraries, demands substantial financial and temporal investments; for parallel dual-target screening, experimental costs and workloads escalate nearly twofold. While molecular docking-based virtual screening has improved screening efficiency to a degree, it struggles to navigate the exponentially expanding chemical space. Against this backdrop, how to efficiently traverse ultra-large-scale chemical space and simultaneously identify molecules with potential activity against two targets within acceptable computational budgets has become a central challenge in computational drug discovery.

Recent breakthroughs in artificial intelligence have offered a revolutionary solution to this predicament. Deep learning models, by projecting molecular structures and protein pockets into latent vector spaces, convert complex physicochemical interactions into efficient similarity retrieval problems, achieving million-fold improvements in screening throughput. AI virtual screening approaches built on deep contrastive learning map protein pockets and small molecules into a unified vector space, enabling parallel scoring of billion-scale compound libraries through a single forward pass and compressing what would conventionally require centuries of docking computation into mere hours [15]. Building upon this foundation, deep generative docking models have further transcended the paradigmatic constraints of traditional docking. DiffDock, representative of diffusion probabilistic models, learns the conformational distribution of ligand–protein binding through a stepwise denoising process, achieving order-of-magnitude acceleration in docking speed while preserving high accuracy [16]. Unlike conventional docking software that exhaustively enumerates conformations and relies on empirical scoring functions, DiffDock learns binding pose distributions directly from data, demonstrating outstanding performance in docking accuracy benchmarks. These advances have rendered it feasible to identify molecules possessing concurrent inhibitory activity against both LDHA and BRD4 directly from ultra-large-scale compound libraries.

Furthermore, recent advances in AI-driven docking have produced several alternative frameworks, including convolutional neural network–based rescoring methods [17] and graph-based scoring architectures [18]. Unlike CNN-based post-docking rescoring approaches that rely on predefined poses, DiffDock employs a diffusion probabilistic framework to directly learn binding pose distributions through iterative denoising, enabling generative sampling of ligand conformations within protein pockets. This generative strategy reduces dependence on exhaustive conformational enumeration and empirical scoring functions, making it particularly suitable for ultra-large-scale virtual screening campaigns where computational efficiency and pose diversity are critical.

In the domain of natural product-derived drug discovery, natural product libraries have attracted considerable attention owing to their structural diversity and biocompatibility. As a preeminent wellspring of drug discovery, natural products have contributed the core scaffolds or parent structures of over 50% of approved small-molecule therapeutics, and their unique chemical space offers excellent complementarity to synthetic compound collections [19]. This advantage is especially pronounced in multi-target drug discovery, where the evolutionarily optimized, multi-pharmacophore scaffolds of natural products inherently provide a structural foundation for interacting with diverse biological targets. The COCONUT (COlleCtion of Open Natural prodUcTs) database aggregates globally reported natural products encompassing over 695,000 compounds spanning plant, microbial, and marine origins, representing the largest open-access natural product resource to date [20]. Beyond providing two-dimensional structures and physicochemical properties, COCONUT integrates multidimensional information including source organisms, geographic distributions, and primary literature, facilitating post-screening candidate traceability. Marine natural products occupy a distinctive chemical space characterized by unique halogenation, ether ring, and macrolide structural features, frequently exhibiting novel mechanisms of action and favorable druggability. The Comprehensive Marine Natural Products Database (CMNPD) catalogs approximately 47,000 marine-derived compounds with systematic bioactivity annotations and taxonomic information [21]. Studies have shown that marine natural products possess unique advantages in antitumor and immunomodulatory applications, providing a vital resource for exploring novel scaffolds with dual metabolic enzyme inhibition and epigenetic regulatory activities [22].

At the computational chemistry level, molecular dynamics (MD) simulations and binding free energy calculations serve as indispensable tools for evaluating the stability and affinity of protein–ligand interactions. Compared to static docking, MD simulations capture conformational dynamics of both protein and ligand, solvent effects, and entropic contributions, more faithfully reflecting binding behavior under physiological conditions. Empirical force fields such as AMBER99SB-ILDN have been extensively benchmarked and validated for accurate description of protein systems [23]. The MM-GBSA (Molecular Mechanics–Generalized Born Surface Area) method, by combining molecular mechanics energies with implicit solvation models, yields binding free energy estimates consistent with experimental trends at modest computational cost [24]. Its energy decomposition functionality further identifies binding hotspot residues, providing molecular-level explanations for how candidate molecules simultaneously accommodate the LDHA catalytic pocket and BRD4 bromodomain and guiding subsequent structural optimization. Quantum chemical calculations, in turn, illuminate molecular reactivity and interaction fundamentals at the electronic level. Density functional theory (DFT) methods can compute frontier orbital distributions, electrostatic potential surfaces, and local electrophilic/nucleophilic indices at reasonable accuracy, facilitating understanding of electronic complementarity between candidate molecules and both target binding sites [25]. The efficient algorithms of quantum chemistry software such as ORCA enable DFT calculations on systems of several hundred atoms to be completed on personal workstations, providing convenient access to in-depth analysis of dual-target binding mechanisms [26].

Grounded in the foregoing rationale, this study employs an integrated AI virtual screening and computational cascade strategy to undertake a de novo discovery campaign for dual-target inhibitors against two key mediators of HCC immune evasion—LDHA and BRD4. This work aims to identify novel anti-HCC lead compounds capable of concurrently suppressing LDHA metabolic activity and BRD4 epigenetic reading function, thereby synergistically reducing lactate/Kla levels and downregulating B7-H3 immune checkpoint expression while simultaneously establishing a generalizable methodological paradigm for multi-target synergistic inhibitor discovery. An overview of the study workflow is presented in the Graphical Abstract.

## 2. Results

### 2.1. Active Binding Pocket Identification

**Figure 1:**
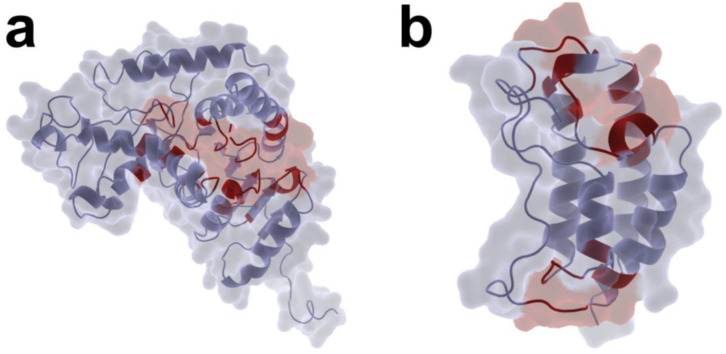
Predicted ligand-binding pockets of LDHA and BRD4 identified by UniSite. (**a**) Potential active binding pockets of LDHA with drug scores ≥ 0.8, mapped onto the protein surface. (**b**) Potential active binding pockets of BRD4 with drug scores ≥ 0.8, mapped onto the protein surface. Red regions indicate the predicted binding pockets located within deep clefts between structural domains, exhibiting moderate hydrophobicity and polar surface distributions characteristic of typical small-molecule binding cavities. Protein structures were obtained from the PDB database (LDHA: PDB ID 4JNK; BRD4: PDB ID 7ZE6) and visualized using PyMOL (v3.1.6.1).

Given the homotetrameric architecture of LDHA, in which all four polypeptide chains share identical sequences, a single protomer was extracted for pocket prediction using the UniSite model, which identified 19 putative binding pockets. For BRD4, the UniSite model recognized 25 potential binding sites (Table S1). Pockets with druggability scores exceeding 0.8 were retained for subsequent virtual screening. Visualization revealed three high-confidence active pockets on LDHA (Figure 1a) and five on BRD4 (Figure 1b), with red-shaded regions denoting the predicted binding cavities. These pockets reside within deep inter-domain clefts and exhibit balanced hydrophobic and polar surface distributions characteristic of canonical small-molecule binding sites.

### 2.2. Construction of the Virtual Screening Library

The initial natural product ligand library was subjected to drug-likeness evaluation. Applying Lipinski’s rule of five (RO5) and quantitative estimate of drug-likeness (QED) scoring to approximately 700,000 small molecules yielded 37,280 compounds from the terrestrial natural product collection and 47,450 from the marine natural product subset that met the drug-likeness criteria.

### 2.3. AI-Assisted Virtual Screening

The 37,280 terrestrial and 47,450 marine natural product ligands were docked against both target proteins using the deep learning-based docking engine DiffDock. Results were ranked by confidence score, and top-scoring molecules exhibiting high predicted affinity for each target were extracted. Intersection of the LDHA and BRD4 hit lists identified six compounds from the terrestrial natural product library and five from the marine collection—totaling 11 dual-target candidates (Table 1). These compounds were subsequently subjected to semi-flexible molecular docking with Quick Vina (version 1.2.7) for binding energy validation (Table 2). Following rigorous evaluation, CNP0038114.1 and CMNPD16582 were designated as the core lead candidates, each demonstrating superior binding performance against both LDHA and BRD4.

### 2.4. Binding Mode Analysis

To precisely elucidate the binding modes and pharmacophoric features of the lead compounds, comprehensive non-covalent interaction profiling of the optimal docked complex conformations was performed using the PLIP algorithm, which systematically extracted and quantified hydrogen bond networks, hydrophobic contacts, electrostatic interactions, and π–π stacking effects within the ligand–receptor interface. In the interaction maps, ligands and key interacting residues are highlighted as stick models, with hydrogen bonds and π–π stacking geometries explicitly displayed. Receptor electrostatic potential surfaces were additionally mapped to contextualize the docking relationships.

**Figure 2:**
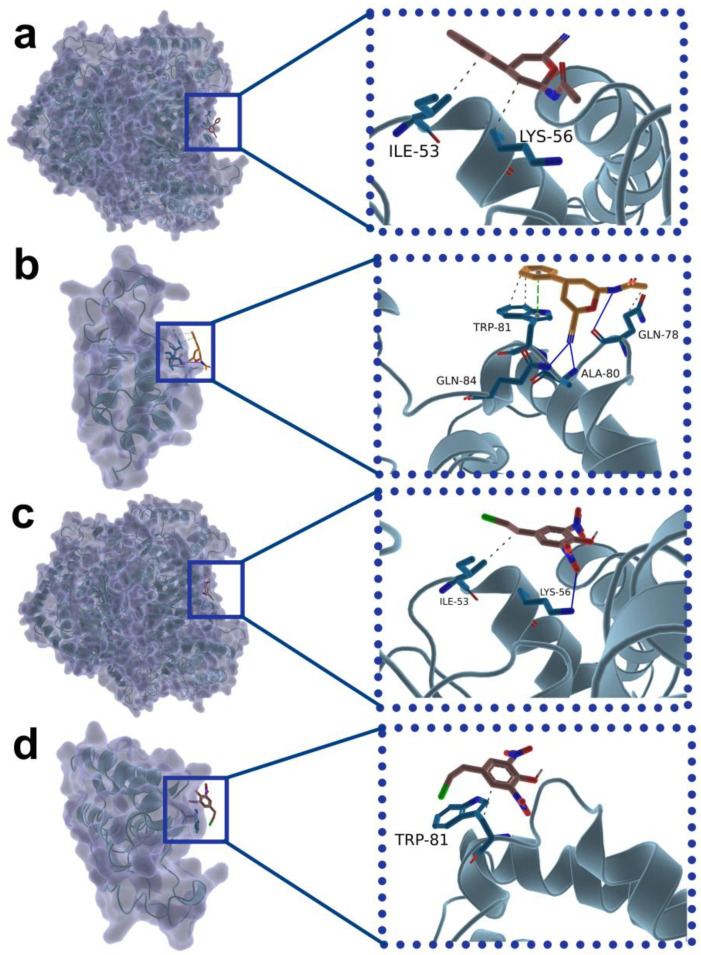
Molecular interaction analysis of the two candidate compounds with LDHA and BRD4. Non-covalent interactions between the ligands and target proteins were analyzed using PLIP and visualized in PyMOL. Ligands and key interacting residues are displayed as stick models; hydrogen bonds are shown as blue lines, hydrophobic interactions as gray dashed lines, and π–π stacking interactions as green dashed lines. The electrostatic potential surface of the receptor protein is shown to illustrate the binding context. (**a**) CNP0038114.1–LDHA complex. (**b**) CNP0038114.1–BRD4 complex. (**c**) CMNPD16582–LDHA complex. (**d**) CMNPD16582–BRD4 complex.

Analysis revealed that CNP0038114.1 establishes two hydrophobic contacts with LDHA residues ILE53 and LYS56 (Figure 2a), while engaging BRD4 through three hydrogen bonds, three hydrophobic interactions, and one π–π stacking interaction involving residues TRP81, GLN84, ALA80, and GLN78 (Figure 2b). CMNPD16582 forms one hydrophobic interaction and one hydrogen bond with LDHA residues ILE53 and LYS56. This binding site is located distal to the canonical catalytic triad (Arg105, His192, Asp194), consistent with an allosteric rather than orthosteric mechanism of inhibition. (Figure 2c). And establishes one hydrophobic contact with BRD4 residue TRP81 (Figure 2d). These interaction profiles indicate that both small molecules form thermodynamically viable docking complexes with both target proteins.

### 2.5. Molecular Dynamics Simulations

Having established CNP0038114.1 and CMNPD16582 as prospective dual-target LDHA/BRD4 inhibitors through the preceding screening pipeline, we proceeded with 200 ns all-atom MD simulations to rigorously assess binding stability.

**Figure 3:**
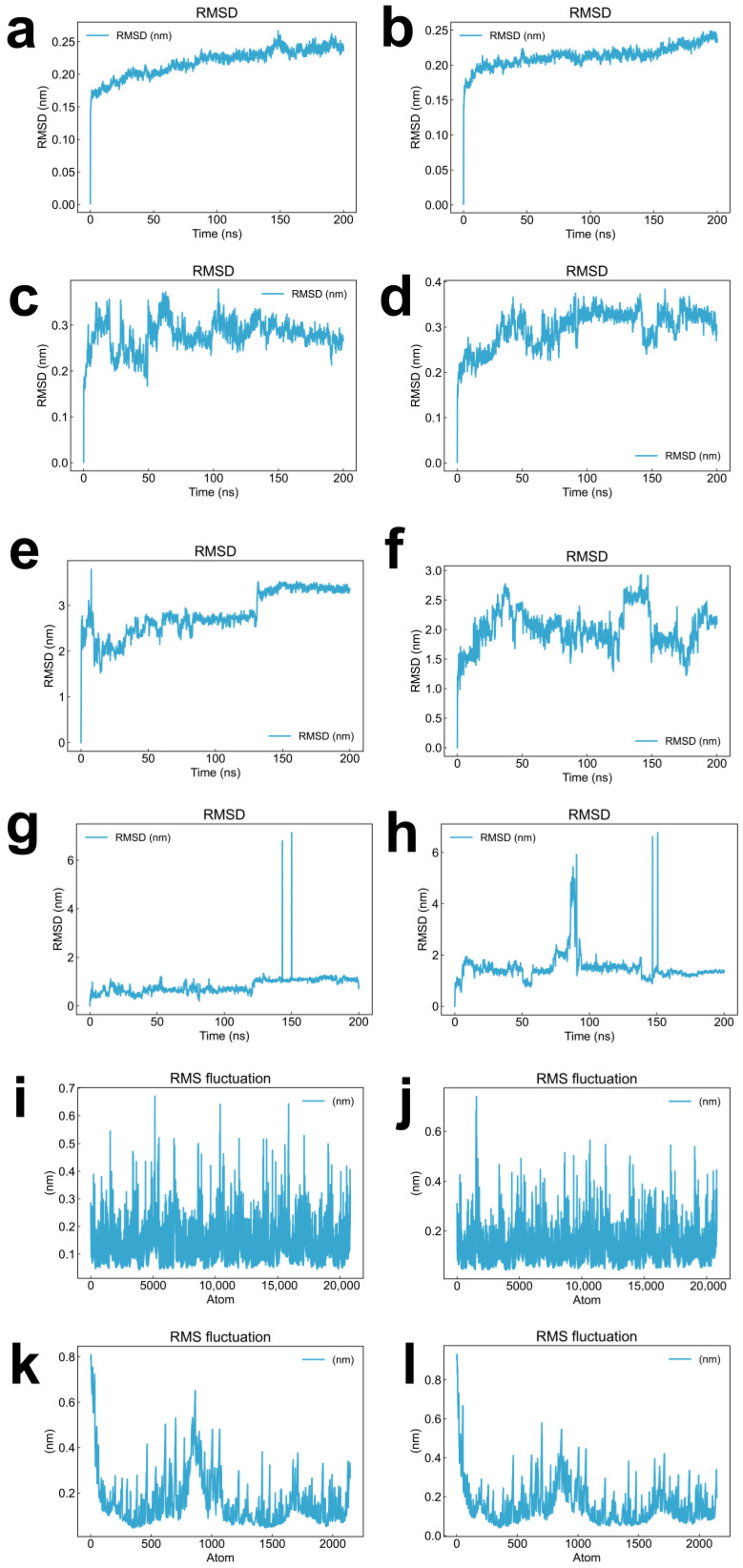
Molecular dynamics simulation analysis of protein–ligand complexes over 200 ns. (**a**–**d**) Root-mean-square deviation (RMSD) of protein backbone atoms as a function of simulation time for: (**a**) CNP0038114.1–LDHA, (**b**) CMNPD16582–LDHA, (**c**) CNP0038114.1–BRD4, and (**d**) CMNPD16582–BRD4 complexes. (**e**–**h**) RMSD of ligand backbone atoms as a function of simulation time for: (**e**) CNP0038114.1–LDHA, (**f**) CMNPD16582–LDHA, (**g**) CNP0038114.1–BRD4, and (**h**) CMNPD16582–BRD4 complexes. (**i**–**l**) Root-mean-square fluctuation (RMSF) per residue for: (**i**) CNP0038114.1–LDHA, (**j**) CMNPD16582–LDHA, (**k**) CNP0038114.1–BRD4, and (**l**) CMNPD16582–BRD4 complexes.

Root-mean-square deviation (RMSD) trajectories of protein backbone atoms revealed that both LDHA complexes—with CNP0038114.1 (Figure 3a) and CMNPD16582 (Figure 3b)—exhibited pronounced conformational stability throughout the simulation. The BRD4 complexes with CNP0038114.1 (Figure 3c) and CMNPD16582 (Figure 3d) achieved convergence and maintained satisfactory stability in the latter stages of the trajectories, collectively indicating that both target proteins preserve their structural integrity upon ligand binding.

To directly assess whether the candidate ligands remained stably bound throughout the entire simulation timescale, we computed the root-mean-square deviation (RMSD) of ligand heavy atoms relative to their initial docked conformations (Figure 3e–h). For the LDHA complexes, the CNP0038114.1 ligand RMSD increased rapidly during the initial equilibration phase, followed by stabilization throughout the remainder of the simulation (Figure 3e), indicating conformational adjustment within the binding pocket and subsequent sustained occupancy. In contrast, CMNPD16582 in complex with LDHA exhibited larger fluctuations, suggesting its stability may be weaker than that of CNP0038114.1 (Figure 3f). For the BRD4 complexes, CNP0038114.1 displayed relatively stable ligand RMSD throughout the simulation, and although two larger fluctuations occurred, there was no systematic drift toward higher values, consistent with dynamic yet stable binding within the bromodomain pocket (Figure 3g). CMNPD16582–BRD4 exhibited similar results, with the overall structure remaining relatively stable (Figure 3h). Notably, none of the four systems showed progressive, unidirectional increases in ligand RMSD that would signal complete unbinding or migration away from the binding site.

Root-mean-square fluctuation (RMSF) profiling demonstrated that LDHA residues in complex with CNP0038114.1 (Figure 3i) and CMNPD16582 (Figure 3j) exhibited minimal positional fluctuation, signifying sustained structural rigidity during the simulations. For BRD4 complexes with CNP0038114.1 (Figure 3k) and CMNPD16582 (Figure 3l), elevated fluctuations were confined to approximately the first 1000 atoms, likely corresponding to intrinsically flexible loop regions of the protein.

**Figure 4:**
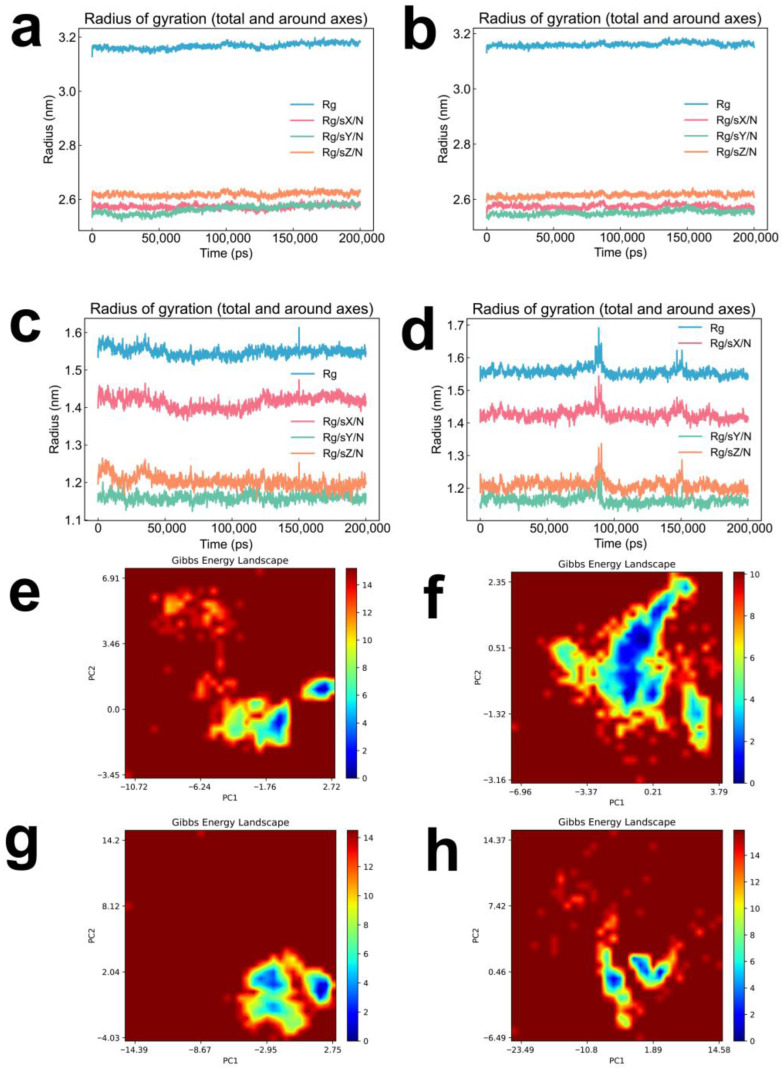
Radius of gyration (Rg) and Gibbs free energy landscape (FEL) analyses. (**a**–**d**) Radius of gyration as a function of simulation time for: (**a**) CNP0038114.1–LDHA, (**b**) CMNPD16582–LDHA, (**c**) CNP0038114.1–BRD4, and (**d**) CMNPD16582–BRD4 complexes. (**e**–**h**) Two-dimensional Gibbs free energy landscape plots projected onto the first two principal components (PC1 and PC2) derived from principal component analysis (PCA) for: (**e**) CNP0038114.1–LDHA, (**f**) CMNPD16582–LDHA, (**g**) CNP0038114.1–BRD4, and (**h**) CMNPD16582–BRD4 complexes. Blue regions represent energy minima corresponding to the most stable conformational states of the ligand–protein complexes.

Radius of gyration (Rg) analysis revealed minimal oscillations across all four complexes (Figure 4a–d), indicating that the protein architectures remained compact and structurally cohesive upon ligand binding. Principal component analysis (PCA)-derived Gibbs free energy landscape (FEL) plots (Figure 4e–h) further illustrated the energy distribution across the first two principal components, with deeper blue regions denoting lower energy minima and hence more thermodynamically stable conformational states of the ligand–protein assemblies. It is worth noting that the blue area in Figure 4f is significantly wider than that of other systems, suggesting that the CMNPD16582–LDHA system has a more flexible conformation, while the CMNPD16582–BRD4 system (Figure 4h) exhibits a more concentrated dark blue area, reflecting the close geometric and energy complementarity between CMNPD16582 and BRD4.

**Figure 5:**
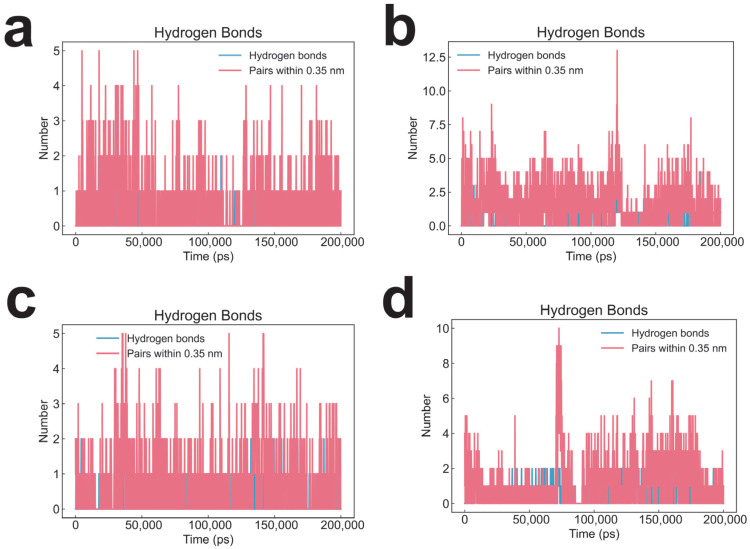
Hydrogen bond analysis during 200 ns molecular dynamics simulations. Number of intermolecular hydrogen bonds between the ligand and protein as a function of simulation time for: (**a**) CNP0038114.1–LDHA, (**b**) CMNPD16582–LDHA, (**c**) CNP0038114.1–BRD4, and (**d**) CMNPD16582–BRD4 complexes.

Intermolecular hydrogen bond analysis throughout the simulations revealed that the LDHA–CNP0038114.1 complex (Figure 5a) formed fewer hydrogen bonds compared to the LDHA–CMNPD16582 complex (Figure 5b). Similarly, CNP0038114.1 (Figure 5c) engaged in fewer hydrogen bond interactions with BRD4 than did CMNPD16582 (Figure 5d). These observations suggest that CMNPD16582 may possess superior potential binding competence toward both targets relative to CNP0038114.1.

### 2.6. Results of Binding Free Energy Calculations

**Figure 6:**
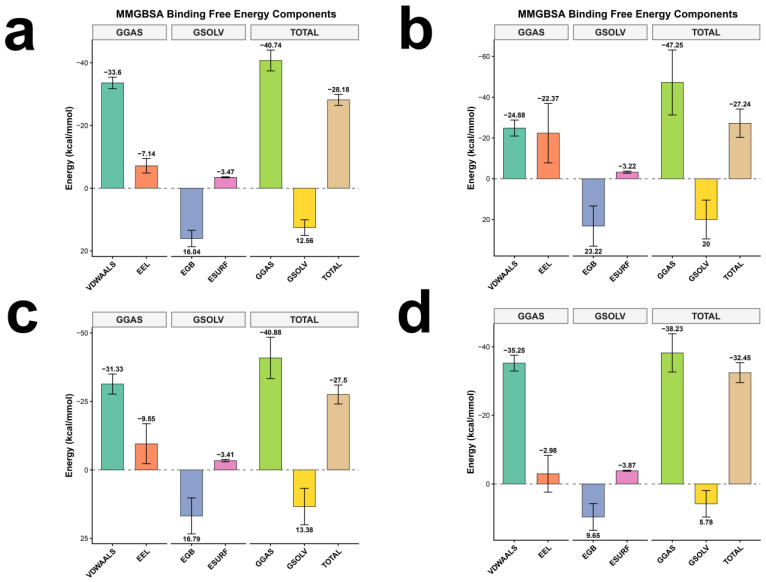
MM-GBSA binding free energy decomposition for the candidate compound–target complexes. Energy decomposition profiles showing the contributions of van der Waals interactions (ΔVDWAALS), electrostatic interactions (ΔEEL), polar solvation energy (ΔEGB), and nonpolar solvation energy (ΔESURF) for: (**a**) CNP0038114.1–LDHA (ΔG = −28.18 kcal/mol), (**b**) CMNPD16582–LDHA (ΔG = −27.24 kcal/mol), (**c**) CNP0038114.1–BRD4 (ΔG = −27.50 kcal/mol), and (**d**) CMNPD16582–BRD4 (ΔG = −32.45 kcal/mol). Across all four systems, van der Waals interactions served as the predominant thermodynamic driving force for binding. Binding free energies were calculated using gmx_MMPBSA with the GB-OBC model (igb = 5) and a salt concentration of 0.15 M.

To systematically evaluate the binding affinities of the candidate compounds toward both targets, MM-GBSA binding free energies were computed for CNP0038114.1 and CMNPD16582 in complex with LDHA and BRD4, accompanied by energy component decomposition (Figure 6a–d).

Energy decomposition consistently revealed that van der Waals interactions (ΔVDWAALS) constitute the predominant thermodynamic driving force across all four systems, with contributions ranging from −24.88 to −35.25 kcal/mol, reflecting favorable shape complementarity between the candidate molecules and both target binding pockets. Electrostatic contributions (ΔEEL), while energetically favorable in the gas phase (−2.98 to −22.37 kcal/mol), were substantially offset by polar desolvation penalties (ΔEGB = +9.65 to +23.22 kcal/mol), rendering the net polar contribution unfavorable for binding in all systems—a thermodynamic signature consistent with the majority of protein–ligand interaction systems. The nonpolar solvation term (ΔESURF = −3.22 to −3.87 kcal/mol) provided modest yet consistently favorable contributions attributable to the hydrophobic effect.

From a comparative dual-target perspective, CNP0038114.1 exhibited balanced affinities toward LDHA and BRD4, with total binding free energies of −28.18 and −27.50 kcal/mol, respectively (Figure 6a and Figure 6c), the former characterized by a more pronounced van der Waals contribution (−33.60 vs. −31.33 kcal/mol). CMNPD16582 displayed differential target preference, yielding binding free energies of −27.24 kcal/mol for LDHA and −32.45 kcal/mol for BRD4 (Figure 6b,d). The superior BRD4 affinity was primarily attributable to enhanced van der Waals complementarity (ΔVDWAALS = −35.25 kcal/mol, the most negative across all four systems). Notably, the CMNPD16582–BRD4 system exhibited diminished electrostatic contributions (ΔEEL = −2.98 kcal/mol) and desolvation penalties (ΔEGB = +9.65 kcal/mol), suggesting that this ligand engages BRD4 predominantly through hydrophobic rather than polar interactions—a binding mode consistent with the largely hydrophobic character of the BD1 bromodomain acetyl-lysine recognition cavity.

Collectively, the total binding free energies of both candidate compounds across both targets ranged from −27.24 to −32.45 kcal/mol, indicating favorable thermodynamic stability for all four complexes and further substantiating the viability of CNP0038114.1 and CMNPD16582 as LDHA/BRD4 dual-target lead compounds.

### 2.7. Density Functional Theory Analysis

To interrogate the chemical reactivity and druggability potential of the candidate compounds at the electronic structure level, DFT calculations were performed on CNP0038114.1 and CMNPD16582, focusing on frontier molecular orbital (FMO) energies and spatial distribution characteristics.

**Figure 7:**
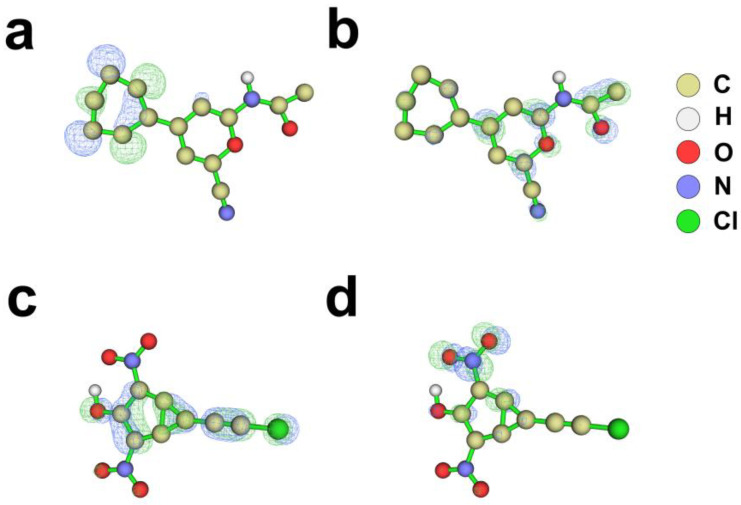
Frontier molecular orbital (FMO) analysis of the candidate compounds by DFT calculations. HOMO and LUMO isosurface plots for: (**a**) HOMO of CNP0038114.1. (**b**) LUMO of CNP0038114.1. (**c**) HOMO of CMNPD16582. (**d**) LUMO of CMNPD16582.

CNP0038114.1 exhibited a HOMO energy of −7.044 eV, a LUMO energy of −4.327 eV, and a HOMO–LUMO energy gap (ΔE) of 2.718 eV. This comparatively narrow gap indicates pronounced electron-transition capacity and chemical reactivity, favorable for charge-transfer interactions with the target proteins, though it simultaneously implies a potential risk of metabolic lability in vivo. Regarding spatial orbital topology, the HOMO was predominantly localized over the phenyl ring region (Figure 7a), where the enriched π-electron density designates this moiety as the principal electron-donating site, capable of engaging in cation–π interactions or π–π stacking with electron-deficient residues within the LDHA or BRD4 active pockets—corroborating the binding mode analysis findings. The LUMO was primarily distributed across the dihydropyran ring and the acetamido moiety (Figure 7b), indicating the propensity of these regions to accept electrons and serve as hydrogen bond acceptor sites for interaction with hydrogen bond donor residues of the target proteins. CMNPD16582 displayed a HOMO energy of −7.917 eV, a LUMO energy of −3.095 eV, and an energy gap of 4.822 eV. This moderate-to-large gap suggests a favorable equilibrium between chemical reactivity and stability, with potentially superior metabolic robustness compared to CNP0038114.1. Notably, the substantially lower HOMO energy of CMNPD16582 (−7.917 eV) relative to CNP0038114.1 (−7.044 eV) reflects the powerful electron-withdrawing influence of the two ortho-nitro substituents, which render the entire molecule electron-deficient with attenuated electron-donating capacity. The HOMO was distributed across the benzene ring, distorted phenolic hydroxyl, and partially extended to the chloropropyl domain (Figure 7c); the distorted phenolic hydroxyl oxygen, although significantly weakened by the flanking nitro groups, remains the sole electron-donating moiety capable of functioning as a hydrogen bond donor. The HOMO distribution on the chloropropyl chain suggests potential contributions through hydrophobic contacts and van der Waals interactions. The LUMO was highly concentrated on one nitro group (Figure 7d), identifying this site as the principal electrophilic reactive center capable of charge-transfer interactions with electron-rich protein residues.

## 3. Discussion

This study addressed the metabolic–epigenetic cooperative network underlying HCC immune evasion, targeting LDHA and BRD4—two critical nodes situated at the metabolic supply terminus and transcriptional activation terminus of the “lactate–Kla–B7-H3” immunosuppressive axis, respectively. We successfully established and applied an integrated AI-driven computational cascade workflow for de novo dual-target inhibitor discovery. From ultra-large-scale natural product libraries, two lead compounds with dual-target binding potential—CNP0038114.1 (N-[(2R,4R)-6-cyano-4-phenyl-3,4-dihydro-2H-pyran-2-yl]acetamide) and CMNPD16582 (2-(4-hydroxy-3,5-dinitrophenyl)-ethyl chloride)—were efficiently identified, and their binding modes, dynamic stability, and electronic structure foundations were systematically characterized. Beyond furnishing candidate molecules for LDHA/BRD4-targeted anti-HCC drug development, this work validates a novel methodological paradigm extensible to other multi-target synergistic inhibition scenarios.

The central innovation of this study resides in the integration and cascading of complementary methodologies. Conventional structure-based virtual screening typically confronts dual bottlenecks: insufficient computational throughput when navigating the exponentially expanding chemical space and elevated false-positive rates inherent to single-tier screening hierarchies. For dual-target screening, these challenges are compounded, as traditional approaches necessitate independent screening against each target followed by hit intersection, effectively doubling the workload. Our multi-tiered cascade strategy addresses these limitations by evaluating candidate druggability across multiple physical scales, systematically attenuating false-positive rates at each successive tier.

To surmount the scalability barrier confronting traditional docking with large chemical libraries, we leveraged DiffDock—a deep generative docking engine predicated on diffusion probabilistic modeling. Unlike conventional docking algorithms that exhaustively enumerate conformations and rely on empirical scoring functions, DiffDock learns binding conformational distributions directly from protein–ligand interaction data through an iterative denoising procedure [16], enabling efficient docking and ranking of 84,730 candidate molecules. In the dual-target screening context, DiffDock rapidly scored candidates against both LDHA and BRD4, with subsequent intersection analysis extracting shared hits, substantially reducing the computational barrier. This deep learning-powered screening paradigm compresses what would conventionally require months of docking computation into tractable timeframes, exemplifying the transformative efficiency gains afforded by AI in multi-target drug discovery.

Concurrently, this study augmented molecular dynamics simulations and MM-GBSA binding free energy calculations with density functional theory (DFT) analysis, extending the investigative perspective from classical mechanics to the quantum electronic structure domain. Although molecular force field-based simulation methods effectively capture the dynamic behavior and thermodynamic trends of protein–ligand binding, they cannot directly delineate the electronic essence of intermolecular interactions—charge transfer, orbital overlap, and polarization effects [27]. DFT calculations, by solving the Kohn–Sham equations, provide first-principles descriptions of electronic distributions, frontier orbital energetics, and global reactivity. In this study, the HOMO/LUMO spatial distributions revealed by DFT exhibited striking concordance with the π–π stacking and hydrogen bonding patterns observed in the binding mode analysis, furnishing quantum-level corroboration of classically derived binding models and substantially elevating the credibility of computational predictions.

The theoretical rationale for selecting LDHA and BRD4 as dual targets derives from their functional convergence within the HCC immunosuppressive network. As the central executor of the Warburg effect, LDHA is ubiquitously overexpressed in HCC, and the copious lactate it generates not only directly blunts effector T cell and NK cell cytotoxicity through microenvironmental acidification [28], but more significantly, lactate serves as a novel epigenetic regulatory metabolite driving histone lysine lactylation—a modification first reported by Zhang et al. in 2019 [5] and now established as a widespread mechanism for epigenetic activation of immunosuppressive gene programs across multiple malignancies. In HCC, elevated H3K18la levels are intimately associated with the establishment of an immunosuppressive microenvironment, indicating that LDHA inhibition to reduce lactate/Kla levels may represent an efficacious strategy for reversing tumor immune suppression.

BRD4, functioning as the BET family’s epigenetic reader, drives transcriptional activation at SE regions by recognizing histone acetylation marks [9]. In HCC, BRD4 has been reported to sustain elevated transcription of B7-H3 (CD276). As a B7 family immune checkpoint molecule, B7-H3 is overexpressed across multiple solid tumors and correlates with adverse prognosis [29], constituting a pivotal molecular shield against immunosurveillance. Accordingly, BRD4 inhibition can directly diminish B7-H3 transcriptional output by attenuating SE activity.

Of particular significance, emerging evidence points to mechanistic crosstalk between LDHA and BRD4. LDHA-driven lactate accumulation, by elevating global Kla levels, can indirectly potentiate the epigenetic activation state of immunosuppressive gene loci, including B7-H3. Furthermore, accumulating data indicate that histone lactylation and acetylation modifications may engage in cross-regulatory interactions at shared genomic loci [12], with BRD4’s bromodomain recognition of acetylation marks potentially modulated by adjacent Kla modification states. This metabolic–epigenetic crosstalk provides a robust biological rationale for the simultaneous targeting of LDHA and BRD4.

The two prioritized lead compounds exhibited distinct yet complementary dual-target binding profiles. The acetamido and cyano functionalities of CNP0038114.1 endow the molecule with rich hydrogen bond donor/acceptor capabilities, potentially underpinning its capacity to simultaneously accommodate the LDHA catalytic pocket and BRD4 bromodomain. Molecular docking and PLIP analyses revealed that CNP0038114.1 establishes multiple interactions with BRD4, including π–π stacking with Trp81 and hydrogen bonding with Gln84; notably, the acetamido carbonyl may mimic the key structural features of the acetylated lysine substrate, consistent with the binding mechanism of established BRD4 inhibitors that achieve bromodomain engagement through acetyl-lysine mimicry [8]. DFT analysis provided electronic structure validation for this binding mode: the LUMO distribution across the acetamido and dihydropyran regions identifies these moieties as competent hydrogen bond acceptor sites for engagement with conserved BRD4 residues.

CMNPD16582 exhibited a markedly stronger binding affinity toward BRD4 (ΔG = −32.45 kcal/mol). MM-GBSA energy decomposition revealed that van der Waals interactions predominated in this complex (ΔVDWAALS = −35.25 kcal/mol), while electrostatic contributions and desolvation penalties were both modest, indicating that binding is achieved principally through hydrophobic complementarity with the BD1 bromodomain cavity—a binding mode congruent with the predominantly hydrophobic character of the acetyl-lysine recognition site [30]. However, it warrants emphasis that nitroaromatic compounds are subject to widespread scrutiny in medicinal chemistry due to their potential genotoxicity risk. Nitro groups may undergo metabolic reduction by nitroreductases to generate electrophilic hydroxylamine or nitroso intermediates capable of forming covalent DNA adducts and eliciting mutagenic effects [31]. The presence of two nitro substituents in CMNPD16582 constitutes a structural alert that demands attention during lead optimization. Viable strategies include bioisosteric replacement with alternative electron-withdrawing groups to preserve the electronic and hydrophobic binding contributions while eliminating genotoxicity liability [32]. While CMNPD16582 demonstrated promising computational binding properties, the presence of two nitro substituents raises legitimate toxicological concerns, as nitro groups constitute well-recognized structural alerts in medicinal chemistry due to their association with potential genotoxicity and mutagenicity risks via nitroreductase-mediated bioactivation. Our PLIP interaction analysis and DFT calculations indicate that these nitro groups function predominantly as passive electronic-withdrawing moieties that modulate the global HOMO/LUMO energetics rather than forming specific directional interactions with binding site residues. This finding suggests that the nitro groups can be replaced with less toxic electron-withdrawing bioisosteres without fundamentally disrupting the binding mode. CMNPD16582 is retained in this study not as a development-ready lead, but as a structurally informative scaffold demonstrating the value of exploring marine natural product chemical space for dual-target discovery and providing a template for rational optimization.

A critical consideration arising from our binding mode analysis is that both CNP0038114.1 and CMNPD16582 engage LDHA primarily through residues ILE53 and LYS56, which are located on the surface of the binding domain rather than within the canonical catalytic pocket defined by Arg105, His192, and Asp194. This spatial segregation suggests an allosteric or non-competitive inhibition mechanism rather than direct orthosteric blockade [33,34]. The ILE53/LYS56 region identified in our study resides within a flexible loop that undergoes conformational rearrangement during the catalytic cycle; ligand binding at this site could plausibly interfere with NADH cofactor binding or perturb the electrostatic network stabilizing the catalytic domain geometry. Allosteric inhibitors offer strategic advantages including reduced susceptibility to competitive displacement by high intracellular pyruvate concentrations and potential for isoform-selective targeting of LDHA over LDHB, thereby mitigating cardiotoxicity risks. However, we emphasize that the current computational evidence does not constitute proof of enzymatic inhibition.

An important structural consideration regarding BRD4 engagement is the inherent conformational flexibility of the bromodomain scaffold itself. The ZA and BC loop regions of the BD1 bromodomain, which flank the acetyl-lysine recognition pocket, are well-documented in crystallographic and NMR studies to exhibit intrinsic flexibility and undergo substantial conformational rearrangement during the binding cycle. These loop elements frequently display elevated B-factors or partial disorder in X-ray crystal structures, indicating that their dynamic behavior is an intrinsic structural property of the bromodomain rather than a consequence of ligand-induced destabilization [35,36]. In our 200 ns MD simulations, the BRD4 complexes exhibited localized RMSFs in these loop regions, consistent with their known structural plasticity and functional role in accommodating diverse ligand scaffolds. This localized flexibility, rather than being indicative of global protein instability or poor ligand binding, reflects the physiological conformational dynamics that enable the bromodomain to recognize and bind multiple acetylated histone substrates with varying sequence contexts.

From a druggability standpoint, the physicochemical properties of both candidates broadly comply with Lipinski’s RO5 and QED drug-likeness criteria, though each presents optimization opportunities. The electronic properties revealed by DFT calculations provide molecular-level rationalization for the binding modes observed in the PLIP and MD analyses. For CNP0038114.1, the HOMO localization on the phenyl ring directly supports the observed π–π stacking interaction with TRP81 in the BRD4 pocket. The narrow HOMO–LUMO gap facilitates charge-transfer interactions with this electron-deficient aromatic residue. For CMNPD16582, the substantially lowered HOMO energy and LUMO localization on the nitro group render the entire molecule highly electron-deficient. This electronic profile is ideally suited for the predominantly hydrophobic BRD4 cavity, where van der Waals interactions dominate and polar electrostatic contributions are minimal. The larger HOMO–LUMO gap suggests reduced reactivity and potentially greater metabolic stability compared to CNP0038114.1, though the genotoxicity liability of the nitro groups remains a primary concern for lead optimization.

The successful identification of dual-target candidates from natural product repositories further corroborates the singular advantages of natural products in multi-target drug discovery. Having undergone billions of years of evolutionary refinement, natural products intrinsically harbor “privileged scaffold” architectures predisposed to engaging diverse biological targets [19]. In this study, the COCONUT and CMNPD databases represent terrestrial and marine natural product chemical spaces, respectively, with minimal structural overlap between the two collections, affording rich chemical diversity for the exploration of dual-target molecules with novel mechanisms of action. Particularly noteworthy is that CMNPD16582, sourced from the marine natural product library, bears distinctive halogenation (chlorine) and nitro substitution patterns exceedingly rare among terrestrial natural products, underscoring the unique attributes of the marine chemical space.

Despite the foregoing advances, several limitations merit candid acknowledgment. First, the workflow is entirely computational and lacks biochemical and cell biological validation. Although the multi-tier computational strategy collectively supports candidate potential from dynamic, thermodynamic, and electronic perspectives, computational activity does not equate to biological activity. Whether the candidates genuinely inhibit LDHA enzymatic function and BRD4 acetyl-lysine recognition in vitro, whether they reduce lactate levels and Kla modification in HCC cells, and whether they downregulate B7-H3 expression and potentiate immune cell-mediated killing all await experimental confirmation.

Second, the DiffDock and MM-GBSA methodologies each harbor intrinsic limitations. As a learning-based docking model, DiffDock’s performance may be influenced by training data biases, potentially compromising accuracy for novel or atypical binding modes [37]. The MM-GBSA method, while computationally efficient and suitable for large-scale ranking, provides relatively crude entropy estimates and is sensitive to force field parameter and solvent model selection; the absolute binding free energy values obtained should not be directly equated with experimentally determined affinities [38].

It is important to contextualize the QuickVina docking scores reported in Table 2 within the discovery-oriented objectives of this study. The absolute binding energies are admittedly modest and would not typically be considered indicative of high-affinity binding in isolation. However, we emphasize that our computational workflow was designed for scaffold discovery and ranking from ultra-large natural product libraries rather than for quantitative affinity prediction. The DiffDock-based primary virtual screening, followed by QuickVina re-scoring, served as a multi-tiered filtration cascade to identify structurally novel candidates capable of simultaneously accommodating both LDHA and BRD4 binding pockets—a dual-target criterion that substantially narrows the hit space. In this context, the docking scores functioned as relative ranking metrics to prioritize compounds for computationally intensive MD simulations and binding free energy calculations, which provide more rigorous assessments of binding stability and affinity. Indeed, the MM-GBSA binding free energies computed from the equilibrated MD trajectories are substantially more favorable than the initial docking scores would suggest, reflecting the limitations of static docking scoring functions in capturing dynamic binding contributions, induced-fit effects, and solvent reorganization. The primary contribution of this work lies in demonstrating a generalizable AI-driven workflow for dual-target natural product screening and in identifying chemically tractable lead scaffolds—CNP0038114.1 and CMNPD16582—that warrant further experimental validation and structure-guided optimization to achieve the affinity levels required for therapeutic development.

Additionally, the per-residue energy decomposition for LDHA revealed contributions from only a limited number of residues (ILE53 and LYS56), which may reflect ligand binding at a relatively shallow surface pocket rather than the catalytic core. The canonical LDHA substrate-binding site involves catalytic residues Arg105, His192, and Asp194 [39], and the binding mode observed herein may represent an allosteric or non-competitive inhibition mechanism whose biological significance warrants elucidation through enzyme kinetic experiments. On the other hand, more detailed mechanistic characterization of the per-residue interaction dynamics would be warranted during subsequent lead optimization campaigns aimed at enhancing binding affinity and selectivity.

Our primary objective was the discovery and initial validation of dual-target scaffolds from ultra-large natural product libraries rather than exhaustive benchmarking against known inhibitors. The internal consistency of our MD results—including RMSD convergence, compact Rg maintenance, sustained hydrogen bonding networks, and favorable MM-GBSA energetics—provides substantial evidence for binding stability, but we acknowledge that direct comparison with apo-protein dynamics and reference compound behavior would offer additional interpretive context. These comparative simulations, alongside experimental enzyme kinetic assays, biophysical binding validation, and cellular functional characterization, represent logical directions for subsequent in-depth studies focused on lead optimization and mechanistic elucidation.

## 4. Materials and Methods

### 4.1. Protein Target Structure Acquisition

Three-dimensional structural coordinates of the target proteins were retrieved from the Protein Data Bank (PDB; https://www.rcsb.org/, accessed on 26 November 2025). The PDB entries used were pdb_00004jnk for LDHA and pdb_00007ze6 for BRD4. Both proteins are from Homo sapiens. Protein structures were preprocessed using PyMOL (version 3.1.6.1). Crystallographic water molecules and heteroatoms were removed. Polar hydrogen atoms were added using the default PyMOL algorithm, which assigns protonation states based on standard physiological pH assumptions for ionizable residues. No further manual adjustment of protonation states was performed. The prepared structures were saved in PDB format for subsequent binding pocket prediction and docking studies.

### 4.2. Binding Pocket Prediction

To delineate potential ligand-binding sites on each target protein, we deployed the UniSite model for automated pocket recognition [40]. UniSite employs a graph neural network architecture trained on experimentally validated protein–ligand complexes from the PDB and UniProt databases. The computational environment was established under a Linux operating system using a PyTorch (Version 2.2.0)-based framework. Input PDB files of the target proteins were preprocessed by removing crystallographic water molecules and adding hydrogen atoms, then transformed into spatial graph representations encoding atomic coordinates and chemical features. Residue-level spatial features (including geometric features, physicochemical features, etc.) were extracted via the physics-aware evolutionary operators of UniSite, and inference was performed using pre-trained model weights to compute binding probability scores across the entire protein surface. High-scoring regions were spatially clustered to determine putative pocket coordinates, and the pocket with the largest volume and highest druggability score was selected as the docking target for subsequent virtual screening.

### 4.3. Small-Molecule Compound Libraries

The ligand databases employed for screening were sourced from COCONUT (https://coconut.naturalproducts.net/, accessed on 8 December 2025), a comprehensive open-access natural product repository. Approximately 695,000 compounds were downloaded. To enhance screening efficiency and hit rates, an initial filtration was applied using Lipinski’s rule of five (RO5) and quantitative estimate of drug-likeness (QED) scoring, with selection criteria requiring no more than one RO5 violation and a QED score > 0.5. Additionally, to explore the distinctive chemical space of marine-derived metabolites, a marine natural product subset was downloaded from the Comprehensive Marine Natural Products Database (CMNPD; https://cmnpd.org/, accessed on 13 December 2025) and subjected to identical drug-likeness filtering criteria. The one-dimensional SMILES representations of these 84,730 filtered compounds were subsequently converted to three-dimensional (3D) structures using the cheminformatics toolkit RDKit. All 3D molecular structures were preprocessed with AutoDock Tools (Version 1.5.7), including the addition of polar hydrogen atoms and Gasteiger charge computation, and saved in the PDBQT format required for docking.

### 4.4. Deep Learning-Based Virtual Screening

High-throughput virtual screening was conducted using DiffDock, a structure-based deep learning docking tool capable of exploring ligand binding poses and conformational space with enhanced accuracy and efficiency. For each compound, the DiffDock model generated docking poses ranked by confidence score. The top-ranked pose (Rank 1) for each compound was retained, with its confidence score serving as the primary evaluation metric. Candidate molecules were selected based on comparative binding energy assessment for downstream detailed analysis.

### 4.5. Visualization and Interaction Analysis of Docking Results

A series of visualization analyses were performed to elucidate the interaction modes between candidate compounds and target proteins. Three-dimensional interaction diagrams of the optimal binding modes predicted by DiffDock were rendered using PyMOL, with particular emphasis on hydrogen bond networks and their corresponding bond lengths. The Protein–Ligand Interaction Profiler (PLIP) was subsequently employed to generate comprehensive interaction maps delineating all non-covalent contacts, including hydrogen bonds, hydrophobic interactions, π-stacking, and salt bridges. Pharmacophore features were manually defined and visualized in PyMOL based on the interaction analysis to elucidate the roles of key functional groups in binding.

### 4.6. System Preparation

Complex systems were initialized from the optimal binding poses predicted by DiffDock. The protein moieties were parameterized using the AMBER99SB-ILDN force field. Ligand topology parameters were generated using Sobtop (version 1.0 dev3.2). Partial atomic charges for the ligands were derived via the restrained electrostatic potential (RESP) method implemented in Multiwfn. Specifically, ligand geometries were first optimized and single-point energy calculations performed at the B3LYP/6-31G level of theory using the ORCA quantum chemistry package (version 5.0.4) to generate electrostatic potential files; RESP charges were then fitted using Multiwfn and subsequently integrated into the Sobtop-generated topology files. Each protein–ligand complex was centered in a cubic simulation box with a minimum distance of 10 Å between the solute surface and box edges and solvated with TIP3P water molecules. Appropriate numbers of Na^+^ or Cl^−^ counterions were added to neutralize the net system charge.

### 4.7. Energy Minimization and Equilibration

To eliminate steric clashes and unfavorable atomic contacts, energy minimization was first performed using the steepest descent algorithm with a maximum force convergence criterion of 1000 kJ/(mol·nm). Subsequently, the system was gradually heated from 0 K to 300 K under the canonical (NVT) ensemble over 100 ps, with position restraints of 1000 kJ/(mol·nm^2^) applied to protein heavy atoms. This was followed by 100 ps of equilibration under the isothermal–isobaric (NPT) ensemble using Berendsen or Parrinello–Rahman pressure coupling to maintain pressure at 1 bar and temperature at 300 K, allowing the system density to equilibrate.

### 4.8. Production Simulation

To assess the dynamic stability and binding behavior of the protein–ligand complexes, unrestrained production MD simulations spanning 200 ns were performed using the GROMACS software package (version 2024.4). An integration time step of 2 fs was employed, with all covalent bonds constrained via the LINCS algorithm. Temperature and pressure were regulated using the v-rescale thermostat and Parrinello–Rahman barostat, respectively. A cutoff of 10 Å was applied for van der Waals interactions, while long-range electrostatic interactions were treated using the Particle Mesh Ewald (PME) method. Trajectory frames were saved every 10 ps for subsequent analysis.

### 4.9. Binding Free Energy Calculations

To quantitatively evaluate ligand–protein binding affinity, binding free energies were computed using the MM-GBSA method. The final 100 frames were extracted from the 200 ns production trajectories using the GROMACS built-in tool, gmx trjconv. Free energy calculations were performed with gmx_MMPBSA. Gas-phase energies (intramolecular and van der Waals contributions) were computed by the sander module using the AMBER99SB-ILDN force field, polar solvation energies were estimated using the GB model (igb = 5, GB-OBC1), and nonpolar solvation energies were approximated using the solvent-accessible surface area (SASA) model. The per-residue energy decomposition functionality was employed to partition the total binding free energy across individual amino acid residues, enabling identification of thermodynamic “hotspot” residues critical for ligand binding.

### 4.10. Density Functional Theory Calculations

To probe the molecular properties and chemical reactivity of the candidate compounds at the electronic level, density functional theory (DFT) calculations were carried out using the ORCA 5.0.4 program package. Molecular geometries were fully optimized under gas-phase conditions at the B3LYP/def2-TZVP level of theory, and harmonic vibrational frequency analyses were performed at the same level to confirm that all optimized structures correspond to genuine local minima on the potential energy surface (characterized by the absence of imaginary frequencies). Based on the optimized geometries, frontier molecular orbital (HOMO and LUMO) distributions and energy gaps were computed. Visualization and analysis of calculated results, including molecular electrostatic potential (MESP) maps and frontier orbital plots, were performed using Multiwfn 3.8_dev.

## 5. Conclusions

This study conducted a systematic computational drug discovery investigation targeting two critical nodes of the “lactate–Kla–B7-H3” immunosuppressive axis in hepatocellular carcinoma: LDHA and BRD4. Beyond providing multi-dimensionally validated lead compounds for the development of dual-acting anti-HCC therapeutics targeting LDHA and BRD4, this work establishes an integrated “AI dual-target primary screening–precision validation–synergistic mechanism elucidation” workflow, offering a generalizable methodological paradigm for the de novo discovery of multi-target synergistic inhibitors. Subsequent efforts should encompass enzyme activity inhibition assays, cellular functional characterization, and immune co-culture systems to validate dual-target inhibitory activity and immune-sensitization effects, complemented by structure-guided optimization based on the delineated binding modes, to advance these lead compounds toward preclinical development.

## Figures and Tables

**Table 1:** Physicochemical properties and drug-likeness profiles of the candidate compounds identified from the integrated virtual screening.

Source	Compound ID	Molecular Formula	InChIKey	MW	AlogP	RB	HBA	HBD	TPSA	Ar. Rings	HA
COCONUT	CNP0111928.0	C12H11N3O2	GGOIWQPOOKIKRW-UHFFFAOYSA-N	229.24	1.25	3	4	1	78.1	2	17
COCONUT	CNP0109136.0	C15H12N2O	CLYBLLLSHHJFIW-UHFFFAOYSA-N	236.27	2.4	1	2	1	56.65	2	18
COCONUT	CNP0038114.1	C14H14N2O2	PMIHZUUKQWXHFO-GXTWGEPZSA-N	242.28	2.06	2	3	1	62.12	1	18
COCONUT	CNP0431611.0	C15H16O6	MPVDQQCJVAESHC-UHFFFAOYSA-N	292.29	2.02	4	6	1	82.06	1	21
COCONUT	CNP0156196.0	C16H14O6	AHYBNBVRJMUCOW-UHFFFAOYSA-N	302.28	1.89	1	6	1	85.97	2	22
COCONUT	CNP0015967.1	C13H14N4	KVASDMAGUYUERI-JTQLQIEISA-N	226.28	1.85	1	3	1	55.71	2	17
CMNPD	CMNPD1885	C9H10Cl2O2	ZLVMQAYISVAUPD-PULIVWKDSA-N	221.08	2.9573	1	2	0	26.3	0	13
CMNPD	CMNPD5219	C9H11NO2S5	LHWKFCLGXFKQPG-UHFFFAOYSA-N	325.52	3.9594	3	8	2	55.48	1	17
CMNPD	CMNPD16582	C8H7ClN2O5	UVECJZMPFCYIGJ-UHFFFAOYSA-N	246.61	1.9899	4	5	1	106.51	1	16
CMNPD	CMNPD26054	C8H10O3	OMYHDCWZPFZMSM-UHFFFAOYSA-N	154.16	1.1405	3	2	1	54.37	0	11
CMNPD	CMNPD29127	C7H8O3	VVBIGJOVPZMWGU-UHFFFAOYSA-N	140.13	0.96224	0	3	1	50.44	1	10

InChIKey, IUPAC International Chemical Identifier hash key, serves as a unique, database-searchable molecular fingerprint for unambiguous compound identification and cross-database deduplication; MW, Molecular weight (Da); ALogP, Calculated octanol–water partition coefficient; RB, Number of rotatable bonds; HBA, Number of hydrogen bond acceptors; HBD, Number of hydrogen bond donors; TPSA, Topological polar surface area (Å^2^); Ar. Rings, Number of aromatic rings; HA, Number of heavy (non-hydrogen) atoms; QED, Quantitative estimate of drug-likeness. ALogP was computed using the Ghose–Crippen–Viswanadhan atom-type approach. Compounds satisfying Lipinski’s rule of five (MW ≤ 500 Da, ALogP ≤ 5, HBA ≤ 10, HBD ≤ 5) and Veber’s criteria (RB ≤ 10, TPSA ≤ 140 Å^2^) are considered to possess favorable oral bioavailability profiles.

**Table 2:** Binding affinities of the top-ranked candidate compounds against LDHA and BRD4.

Source	Compound ID	LDHA BE	BRD4 BE	Mean BE
COCONUT	CNP0015967.1	−3.7	−4.4	−4.05
COCONUT	CNP0038114.1	−4.4	−5	−4.7
COCONUT	CNP0109136.0	−3.6	−4.9	−4.25
COCONUT	CNP0111928.0	−3.4	−4.4	−3.9
COCONUT	CNP0156196.0	−4.1	−4.9	−4.5
COCONUT	CNP0431611.0	−2.7	−4.2	−3.45
CMNPD	CMNPD1885	−3.1	−3.5	−3.3
CMNPD	CMNPD5219	−3.2	−3.7	−3.45
CMNPD	CMNPD16582	−3.2	−4.4	−3.8
CMNPD	CMNPD26054	−2.6	−3.3	−2.95
CMNPD	CMNPD29127	−3.1	−3.7	−3.4

BE, Binding Energy (kcal/mol).

## Data Availability

All data supporting the findings of this study are available within the article and its Supplementary Materials. The protein structures used in this study were retrieved from the Protein Data Bank (https://www.rcsb.org/; accessed on 26 November 2025; PDB IDs: 4JNK for LDHA and 7ZE6 for BRD4). The natural product compound libraries were obtained from the COCONUT database (https://coconut.naturalproducts.net/; accessed on 8 December 2025) and the CMNPD (https://cmnpd.org/; accessed on 13 December 2025).

## Supplementary Materials

The following supporting information can be downloaded at: https://www.mdpi.com/article/10.3390/ph19050736/s1, Table S1: Complete list of predicted binding pockets for LDHA and BRD4 identified by the UniSite model.

## Abbreviations

The following abbreviations are used in this manuscript:

AI	Artificial intelligence
BD1/BD2	Bromodomain 1/Bromodomain 2
BET	Bromodomain and extra-terminal domain
BRD4	Bromodomain-containing protein 4
CMNPD	Comprehensive Marine Natural Products Database
COCONUT	COlleCtion of Open Natural prodUcTs
DFT	Density functional theory
FEL	Free energy landscape
FMO	Frontier molecular orbital
GB	Generalized Born
H3K18la	Histone H3 lysine 18 lactylation
HCC	Hepatocellular carcinoma
HOMO	Highest occupied molecular orbital
Kla	Lysine lactylation
LDHA	Lactate dehydrogenase A
LINCS	Linear Constraint Solver
LUMO	Lowest unoccupied molecular orbital
MD	Molecular dynamics
MESP	Molecular electrostatic potential
MM-GBSA	Molecular mechanics–generalized Born surface area
NK	Natural killer
NPT	Isothermal–isobaric ensemble (constant number, pressure, temperature)
NVT	Canonical ensemble (constant number, volume, temperature)
PCA	Principal component analysis
PDB	Protein Data Bank
PDBQT	Protein Data Bank with partial charge and atom type
PLIP	Protein–Ligand Interaction Profiler
PME	Particle Mesh Ewald
QED	Quantitative estimate of drug-likeness
RESP	Restrained electrostatic potential
Rg	Radius of gyration
RMSD	Root-mean-square deviation
RMSF	Root-mean-square fluctuation
RO5	Rule of five
SASA	Solvent-accessible surface area
SE	Super-enhancer
SMILES	Simplified molecular input line entry system
TME	Tumor microenvironment
